# Transvitreal Retinochoroidal Biopsies of Primary Uveal Melanoma Reveal an Association of Low HLA Class I and High NK Cell Abundance in Low-Risk Disease

**DOI:** 10.1167/iovs.66.2.24

**Published:** 2025-02-07

**Authors:** Kalijn Fredrike Bol, Mark Wilhelmus Dirk Sweep, Michael Douglas Crowther, Mark Antonius Johannes Gorris, Pedro Aroca Lara, Arianna Draghi, Mette Marie Bagger, Carsten Faber, Johannes Textor, Marco Donia, Jens Folke Kiilgaard, Inge Marie Svane

**Affiliations:** 1 Department of Medical Oncology, Radboud University Medical Center, Nijmegen, the Netherlands; 2 Department of Medical BioSciences, Radboud University Medical Center, Nijmegen, the Netherlands; 3 National Center for Cancer Immune Therapy, Department of Oncology, Copenhagen University Hospital, Herlev, Denmark; 4 Data Science, Institute for Computing and Information Sciences, Radboud University, Nijmegen, the Netherlands; 5 Department of Ophthalmology, Rigshospitalet, Copenhagen University Hospital, Copenhagen, Denmark; 6 Department of Clinical Medicine, Faculty of Health and Medical Sciences, Copenhagen University, Copenhagen, Denmark

**Keywords:** uveal melanoma, transvitreal retinochoroidal biopsy, tumor immune microenvironment, tumor-infiltrating lymphocytes

## Abstract

**Purpose:**

Immune cells in primary uveal melanoma (PUM) have mostly been studied in enucleated tissue, thereby precluding material from thinner, low-risk PUM tumors for research purposes. Here, we investigated the feasibility of using tumor tissue acquired by transvitreal retinochoroidal (TVRC) tumor biopsies to study the tumor immune microenvironment and relation to genetic risk class.

**Methods:**

Collected tumor biopsies of 41 patients were tested for genetic aberrations to determine high-risk (*n* = 19), medium-risk (*n* = 12), and low-risk (*n* = 9) tumors and were digested for flow cytometry analysis of immune cell and tumor markers. In addition, 13 patient-matched enucleated tumors were stained using multiplex immunohistochemistry.

**Results:**

Tumor biopsies showed a high variability in the degree of immune infiltration. The tumor-specific lymphocyte infiltration pattern correlated well with the infiltration pattern in patient-matched enucleations. High-risk tumors tended to have a higher abundance of CD8 T cells, which expressed activation markers CD39, CD69, and PD-1, with reduced CD127 expression. In general, low-risk tumors exhibited decreased human leukocyte antigen (HLA) class I expression, coinciding with a higher abundance of natural killer (NK) cells (*P* = 0.0049), indicating a different lymphocyte infiltration pattern between low-, medium- and high-risk tumors.

**Conclusions:**

TVRC biopsies are a suitable and valuable source of PUM tissue for research purposes. An abundance of CD8 T cells was found in high-risk PUM tumors. Further, medium- and low-risk PUM tumors are characterized by decreased HLA class I expression with a concomitant increase in NK cell infiltration as compared to high-risk PUM tumors, correlating with decreased risk of disease recurrence.

The most prevalent primary malignancy of the eye is primary uveal melanoma (PUM).^1^ It mostly originates from melanocytes in the choroid but can also be derived from the iris or ciliary body. In comparison to cutaneous melanoma (CM), PUM is quite rare, with a stable incidence over the last decades of up to 8 cases per million per year in Europe, the United States, and Australia.^2^^–^^4^

In contrast to CM, PUM is characterized by a limited tumor mutational burden but well-defined genetic aberrations.^5^ Generally, driver mutations are present in either *GNAQ* or *GNA11* in PUM.^6^ Common genetic aberrations include monosomy of chromosome 3, gain of 8q, and mutations in *EIF1AX*, *SF3B1*, or *BAP1*.^7^^,^^8^ These factors, as well as gene expression profiling of 15 genes of interest, recently combined with preferentially expressed antigen in melanoma (PRAME) expression, allow classification of tumors according to the risk of developing metastasis.^9^^,^^10^

Regardless of the genetic profile, local treatment is similar across patients. Brachytherapy is the treatment of preference for smaller tumors, whereas larger tumors are often enucleated.^11^ Within 5 years after local treatment, low-risk tumors rarely metastasize, whereas more than half of patients with high-risk tumors develop metastasis, with the liver being the main site of metastasis.^12^^–^^14^ In a metastatic setting, there are very few effective treatments.^15^ With the rise of immunotherapy in metastatic CM, targeting the immune system has been of interest in metastatic uveal melanoma. Tebentafusp, a bispecific T-cell engager targeting gp100 and the T-cell receptor in HLA-A*02:01 patients, has shown improved survival compared to investigators’ choice of treatment.^16^ Unfortunately, radiological responses are low, and the treatment is limited to an human leukocyte antigen (HLA) type and therefore not suitable for many patients. More general treatments such as immune checkpoint inhibition have shown some efficacy in metastatic uveal melanoma, but not nearly as much as in metastatic CM.^17^^–^^21^

To understand the modest effect of immunotherapies in PUM and unravel potential predictive biomarkers, knowledge on the immune system is key. Infiltration of immune cells is correlated with a good prognosis in many cancers, including CM, but PUM is actually one of the few tumor types in which immune infiltration is correlated to a poor prognosis.^22^^–^^26^ It is well established that myeloid cells, especially macrophages, are abundant in PUM, in particular in high-risk tumors.^25^^–^^29^ The most infiltrative subset of macrophages seems to be anti-inflammatory, based on CD163 expression.^26^^,^^27^^,^^30^ Whereas myeloid cells can strongly influence the tumor microenvironment, tumor-infiltrating lymphocytes (TILs) are important mediators in tumor killing, in particular through CD8 T cells and natural killer (NK) cells. Strikingly, TILs in PUM are mostly CD8 dominated and seem to exhibit a pro-inflammatory type II interferon signature in high-risk tumors.^26^^,^^27^^,^^31^ Yet, a deeper cellular understanding of TILs is still quite limited, especially in low-risk tumors.

Immune cells have so far solely been studied in tumors treated by enucleation. Because brachytherapy is currently the most frequent treatment for small and medium-sized tumors, entire patient groups have so far been excluded from translational studies.^32^^,^^33^ Transvitreal retinochoroidal (TVRC) biopsies can be taken as standard of care for genetic testing and could provide an opportunity for more comprehensive investigations of the tumor immune microenvironmental components comprising the whole population of patients with PUM.

Here, we show that immune cell infiltration can be assessed by performing flow cytometry on fresh tumor material obtained with TVRC biopsy. This assessment correlated well with immune cell infiltration determined by multiplex immunohistochemistry (mIHC) on patient-matched enucleated tumor material. Our data validate the findings from prior studies in a more variable patient population, showing more T-cell infiltration in high-risk PUM, dominated by CD8 T cells. By using flow cytometry, we could dive further into the activation status of T cells, thereby detecting increased expression of CD39, CD69, and programmed cell death protein 1 (PD-1) in the tumor, whereas CD127 expression was reduced. In addition, TVRC biopsies enable the investigation of smaller, low-risk tumors, in which NK cells tend to be the major lymphocyte subset, which correlates with the absence of HLA class I expression on these tumor cells. Overall, our study shows the feasibility of using TVRC biopsies to determine the immune infiltrate in PUM, including tumors treated with brachytherapy.

## Materials and Methods

### Patients and Samples

Forty-three patients with clinically diagnosed PUM were enrolled in this study between July 2019 and January 2021. The diagnosis was verified histologically by a pathologist for 42 patients. One patient was excluded due to an HIV infection. Tumor tissue from primary uveal melanomas was obtained through TVRC biopsies using standard 25-gauge vitrectomy equipment (Constellation Vision System 25+ Vitrectomy Total Plus Pak; Alcon, Geneva, Switzerland).^34^ The biopsies were performed at Rigshospitalet (Copenhagen, Denmark) under general anesthesia and taken either shortly before enucleation during the enucleation procedure or directly after plaque placement for brachytherapy. Biopsies were conducted as standard of care for diagnostic testing with additional material taken for this study. Blood samples were collected on the same day as the biopsy. Peripheral blood mononuclear cells (PBMCs) were separated by centrifugation on a Lymphoprep (STEMCELL Technologies, Vancouver, BC, Canada) density gradient and cryopreserved for later use or used directly for flow cytometric analysis. From 13 patients who underwent enucleation, archival formalin-fixed, paraffin-embedded (FFPE) tissue blocks were obtained, which were used for hematoxylin and eosin (H&E) staining and mIHC.

The study was approved by the Ethics Committee of the Capital Region of Denmark (H-18055660) and the Danish Data Protection Agency (VD-2019-28/P-2019-705). Written informed consent was obtained from all patients. The human tissue experiments complied with the guidelines of the ARVO Best Practices for Using Human Eye Tissue in Research.

### Genetic Risk Classification

As standard of care, chromosomal aberrations were identified using fluorescence in situ hybridization, centromeric probes for chromosomes 3 (CEP3 D3Z1) and 8 (CEP8) (Vysis SpectrumOrange probes; Abbott, Abbott Park, IL, USA), and multiplex ligation-dependent probe amplification to evaluate copy number alterations on chromosomes 3p, 3q, 8p, and 8q. The risk factor of tumors was determined according to gain of 8q and monosomy of chromosome 3. The presence of both, one, or neither indicated high, medium, and low risk, respectively. To identify the BRCA-1 associated protein 1 (BAP1) status of tumors, a standard immunohistochemistry (IHC) staining using 1:100 anti-BAP1 (clone C-4, sc-28383; Santa Cruz Biotechnology, Dallas, TX, USA) was performed. This was done on FFPE material for enucleations. For TVRC biopsies, either paraffin-embedded sediments or cytospins were used for BAP1 staining. As standard of care, the staining was evaluated by a specialized eye pathologist of the Department of Pathology at Rigshospitalet.

### Mechanical Dissociation of Tumor Tissue

Tumor tissue that was not used for genetic testing or histology was mechanically dissociated for flow cytometric analysis with the BD Medimachine system (BD Biosciences, Franklin Lakes, NJ, USA) according to the manufacturer's protocol. Briefly, tumor tissue was resuspended in RPMI 1640 Medium (Thermo Fisher Scientific, Waltham, MA, USA) or Dulbecco's phosphate-buffered saline (D-PBS; Sigma-Aldrich, St. Louis, MO, USA). Disposable Medicon capsules with a 50-µm steel mesh with about 100 hexagonal bore-holes framed by six microblades (BD Biosciences) were filled with 1 mL of tumor tissue suspension. The Medicons were inserted into the BD Medimachine and run for 1 minute at a rotation speed of approximately 100 rpm. The aspirated cell suspension was passed through a 70-µm Filcon filter (BD Biosciences). Tumor cell suspensions were used immediately for flow cytometric analysis.

### Flow Cytometry

Tumor cell suspensions and PBMCs were washed in D-PBS and incubated with antibodies in Brilliant Stain Buffer (BD Biosciences) for 20 minutes at 4°C; they were then washed and resuspended in D-PBS. Flow cytometric analysis was performed with a NovoCyte Quanteon (Agilent Technologies, Santa Clara, CA, USA) and analyzed with FlowJo 10. Cells were stained with the monoclonal anti-human antibodies listed in Supplementary Table S1. The gating strategy is shown in Supplementary Figure S1. For analysis of flow cytometry data, several thresholds were implemented. Major immune cell subsets are only reported if CD45^+^ cells exceed 50. The ratios of CD8 and CD4 T cells are reported only if more than 50 CD3 T cells were available. Finally, marker expression on cell subsets is reported only when at least 50 cells of that subset were measured. In addition, regulatory T cells (Tregs) could not be analyzed for all patients (see Supplementary Table S1).

### Multiplex Immunohistochemistry

mIHC was performed on FFPE tissue sections as we have previously described.^35^ Briefly, sections of 4 µm were cut and placed on Epredia Superfrost PLUS adhesion slides (Thermo Fisher Scientific). Slides were then stained on a BOND RX autostainer (Leica Biosystems, Nussloch, Germany), using the order and antibodies listed in Supplementary Table S2. Subsequently, slides were scanned using a PhenoImager HT instrument (Akoya Biosciences, Marlborough, MA, USA). Tissue images were selected using Phenochart 1.1 (Akoya Biosciences), after which corresponding images were spectrally unmixed with inForm 2.4.10 (Akoya Biosciences). The different tissue components of the eye were selected on stitched images by drawing regions in QuPath 0.4.2 (Supplementary Fig. S2A).^36^ H&E staining of an adjacent slide was always used to determine the tissue components in mIHC, confirmed by an ophthalmologist. Immune cells in the tissue were detected with ImmuNet (Supplementary Figs. S2B–S2D).^37^ The machine learning network was improved with manual annotations for this specific dataset. Independent manual annotations were then used for validation of the performance of ImmuNet on our data (Supplementary Fig. S3). For the regions of interest, immune cell counts and areas were extracted and used to determine densities of immune cells.

### Statistical Analysis

Data processing, visualization, and statistical analysis were performed using R 4.1.2 (R Foundation for Statistical Computing, Vienna, Austria). The *P* values in the Table were calculated using an unpaired *t*-test for numerical variables. Categorical variables were tested using a χ^2^ test of independence or with a Fisher's test if expected group sizes were below five. Intraclass correlation coefficients (ICCs) were computed on logit-transformed percentages, using the two-way agreement model within the R package irr 0.84.1. A Fisher's test was performed to test for differences in reaching the threshold for analysis between risk groups, with a Bonferroni correction to compare individual groups. For paired data, Shapiro–Wilk tests were performed to test for normality of the differences between paired groups before applying paired *t*-tests. Differences between risk groups or tumor stage were assessed using analysis of variance (ANOVA), with a post hoc Tukey test when groups were statistically different. However, when one of three groups contained fewer than two observations, an unpaired *t*-test was used instead. In general, data were log transformed prior to statistical analysis when normality could not reasonably be assumed. For survival data, a log-rank test was performed to compute *P* values, as default of the R package survminer 0.4.9. *P* < 0.05 was considered statistically significant.

**Table.** Baseline Characteristics of PUM Patients

	Type of Treatment			
	Brachytherapy (*n* = 27)	Enucleation (*n* = 14)	Total (*N* = 41)	*P* ^*^
Age at diagnosis (y)				0.90
Mean ± SD	68 ± 11	68 ± 13	68 ± 11	
Median (min, max)	70 (45, 84)	71 (47, 88)	70 (45, 88)	
Gender, *n* (%)				1.0
Female	14 (52)	8 (57)	22 (54)	
Male	13 (48)	6 (43)	19 (46)	
Ciliary body involvement, *n* (%)				0.023
No involvement	23 (85)	7 (50)	30 (73)	
Involvement	2 (7.4)	6 (43)	8 (20)	
Only	2 (7.4)	1 (7.1)	3 (7.3)	
AJCC T stage, *n* (%)				0.0019
T1a	11 (41)	1 (7.1)	12 (29)	
T2a	10 (37)	3 (21)	13 (32)	
T2b	3 (11)	0 (0)	3 (7.3)	
T3a	2 (7.4)	3 (21)	5 (12)	
T3b	1 (3.7)	5 (36)	6 (15)	
T3d	0 (0)	2 (14)	2 (4.9)	
Lower basal diameter (mm)				0.036
Mean ± SD	11 ± 2.6	13 ± 3.0	12 ± 2.9	
Median (min, max)	11 (6.5, 17)	14 (7.0, 17)	12 (6.5, 17)	
Thickness (mm)				<0.001
Mean ± SD	3.8 ± 1.9	7.9 ± 2.1	5.2 ± 2.7	
Median (min, max)	3.3 (1.5, 8.0)	8.0 (4.0, 11)	4.5 (1.5, 11)	
Tumor volume (mm^3^)				<0.001
Mean ± SD	190 ± 170	500 ± 250	300 ± 250	
Median (min, max)	140 (24, 710)	500 (130, 910)	170 (24, 910)	
Risk group,^†^ *n* (%)				0.17
Low (disomy 3 and normal 8q)	8 (30)	1 (7.1)	9 (22)	
Medium (monosomy 3 or 8q gain)	8 (30)	4 (29)	12 (29)	
High (monosomy 3 and 8q gain)	10 (37)	9 (64)	19 (46)	
Missing	1 (3.7)	0 (0)	1 (2.4)	

* *P* values represent the results of statistical tests for differences between treatment types.

† Classification according to the CCIT-DK standards.

## Results

### Baseline Characteristics

**Figure 1.**
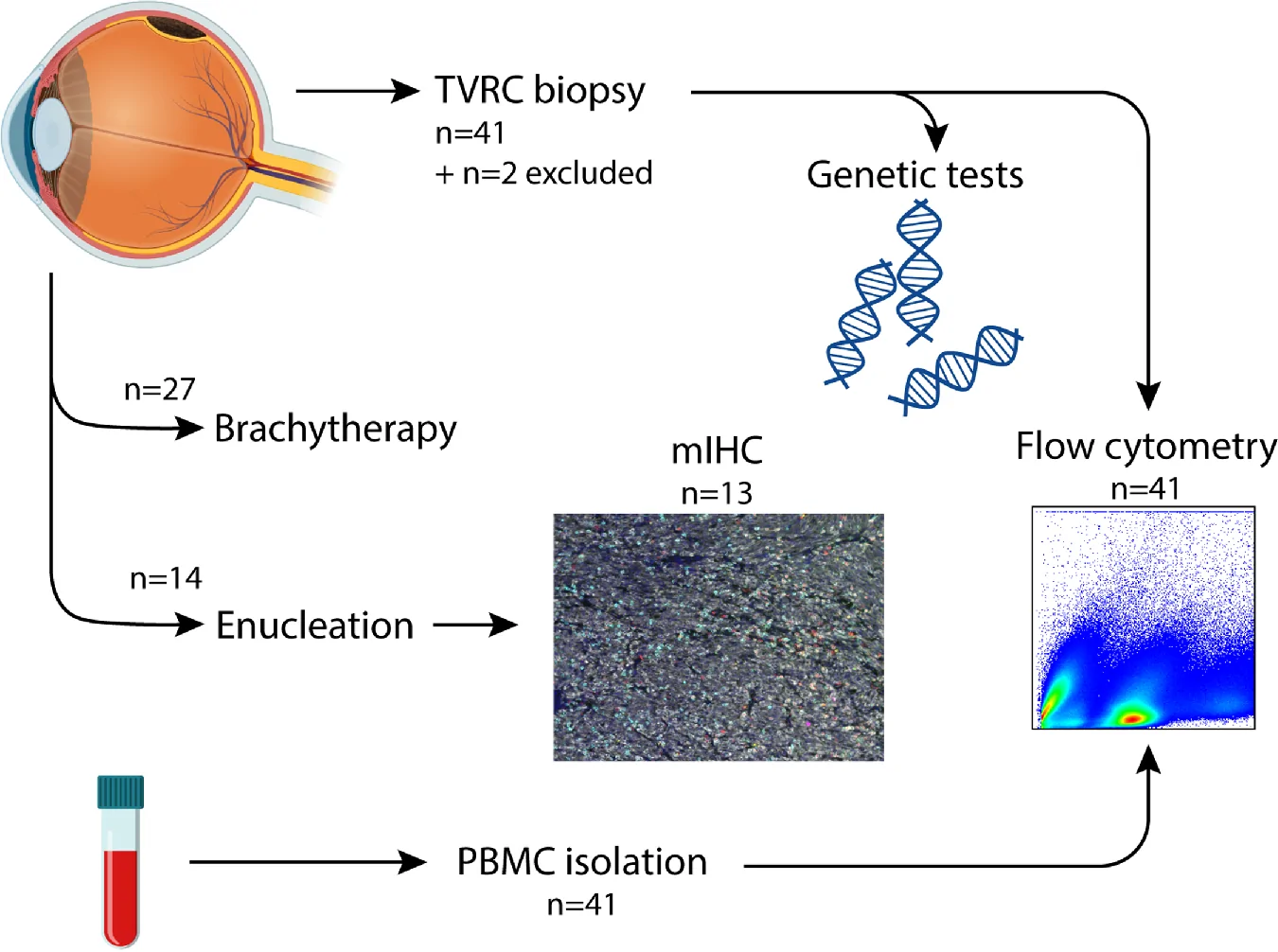
Schematic overview of the study. A total of 43 patients were included, but two had to be excluded. From all patients a TVRC biopsy was taken, which was used for genetic testing and flow cytometry analysis. PBMCs were isolated from peripheral blood as control for flow cytometry. Patients were treated with either brachytherapy or enucleation. From enucleated samples, slices were stained with mIHC.

In total, 41 patients with PUM were included in this study, 14 underwent enucleation as primary treatment, and 27 received brachytherapy. An overview of the study is shown in Figure 1, and patient characteristics are shown in the Table. As expected, differences between patients who received enucleation versus brachytherapy were present. Enucleated tumors often had more ciliary body involvement, higher T staging, and larger lower basal diameter, thickness, and volume of the tumor.

### Tumor-Specific Immune Infiltration Can Be Measured in TVRC Biopsies

**Figure 2.**
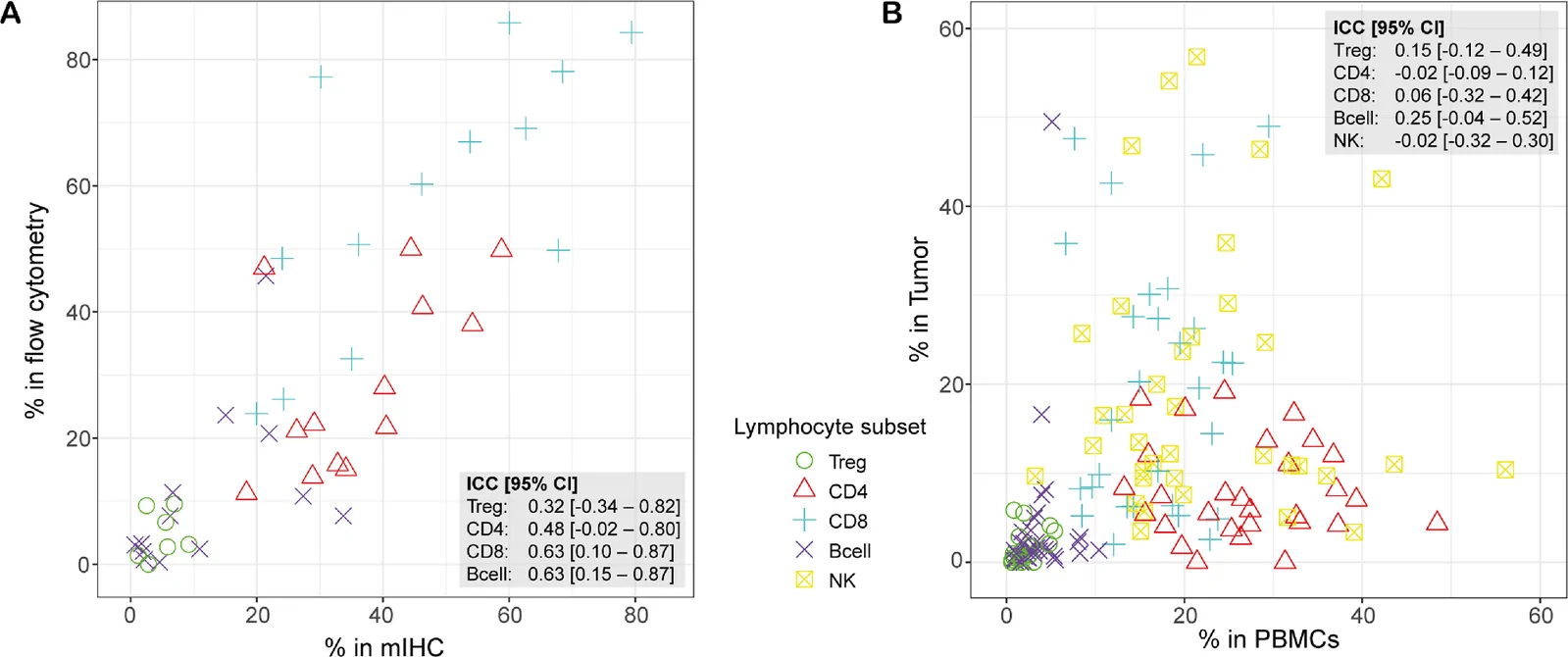
Lymphocyte subsets correlate between paired tumor biopsy and enucleation but not between blood and tumor. (**A**) Percentages of lymphocyte subsets (Tregs, *n* = 7; CD4, *n* = 13; CD8, *n* = 13; B cells, *n* = 13) of all detected B and T lymphocytes between paired tumor biopsy (measured by flow cytometry) and enucleation (measured with mIHC). NK cells were not detected sensitively enough by mIHC and thus were left out of the analysis. (**B**) Percentages of lymphocyte subsets (Tregs, *n* = 17; CD4, *n* = 29; CD8, *n* = 29; B cells, *n* = 36; NK cells, *n* = 36) from all T, NK, and B lymphocytes, in the tumor versus circulating PBMCs, measured by flow cytometry. ICCs with 95% CIs were calculated for each subset.

In previous studies, the immune system in PUM has mostly been investigated using IHC staining of enucleated material, thereby excluding all patients undergoing eye-conserving treatment. Here, we employed flow cytometry on TVRC biopsies to further investigate all subgroups of PUM. First of all, immune cells were detected in all biopsies of both brachytherapy-treated and enucleated patients (Supplementary Fig. S4A). On average, across flow cytometry panels, 30 out of 41 biopsies had more than 50 immune cells (CD45^+^) measured, the threshold defined for further analysis. Low-risk tumors met this threshold less frequently than high-risk tumors (*P* < 0.01 for two out of three panels). For patients treated with enucleation, mIHC was additionally performed on the enucleated tissue. This allowed for comparison of the percentages of TILs between biopsy tissue (flow cytometry) and enucleated tissue (mIHC) from the same patient. Unfortunately, NK cells (CD56^+^) could not be captured by mIHC (Supplementary Fig. S4B), potentially due to the intense CD56^+^ staining of the retina (Supplementary Fig. S4C) and therefore were left out of the main mIHC analysis. Nevertheless, a good agreement on the presence of lymphocytes between mIHC in enucleations and flow cytometry of TVRC biopsies was found (Fig. 2A). ICCs ranged from 0.48 to 0.63 for B cells and T cells (CD4 and CD8). Regulatory T cells (CD127^–^CD25^+^CD4 T cells in flow cytometry and CD8^–^FoxP3^+^ in mIHC) seemed to correlate less, but percentages were also very low. Overall, this finding indicates that flow cytometry is a feasible strategy to study the presence of major TIL subsets in TVRC biopsies of patients who are treated with brachytherapy.

Comparison of immune cell proportions in blood and tumor from the same patients showed no correlation between the composition of TILs and circulating lymphocytes, with near-zero values for most ICCs (Fig. 2B). Within the microenvironment of the eye, unaffected choroid also displayed a different infiltration pattern than that of choroidal tumors (Supplementary Fig. S4D). These data support the observation that infiltration of lymphocytes in PUM is specific to the tumor.

### High-Risk Tumors Are Characterized by T-Cell Infiltration

**Figure 3.**
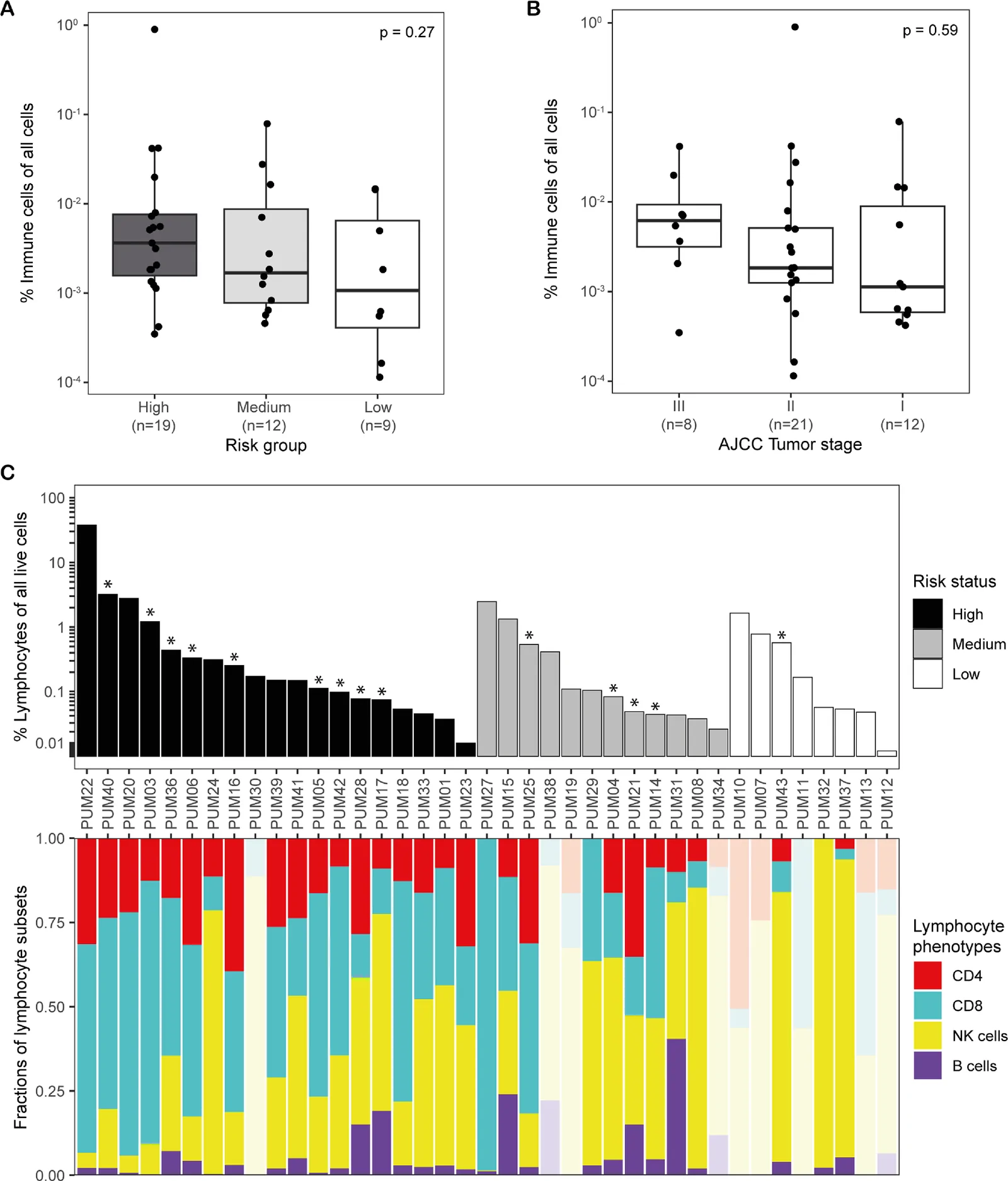
Immune cells and lymphocyte subsets in TVRC biopsies. (**A**, **B**) Box plots showing the percentage of immune cells of all cells, stratified according to risk group (**A**) and AJCC classification of tumor stage (**B**). (**C**) The percentage of lymphocytes from all live cells is shown for each individual patient, stratified according to risk group (*top*). *Asterisks* (*) above bars indicate patients who received enucleation treatment after biopsy. The *lower half* shows the fractions of specific lymphocyte subsets within the total lymphocyte population. *Faded bars* indicate a low number of total lymphocytes (<50) measured by flow cytometry.

Traditionally, PUM tumors are staged in risk groups according to genetic aberrations or by American Joint Committee on Cancer (AJCC) tumor staging based on largest basal diameter, thickness, extraocular extension, and ciliary body involvement. In TVRC biopsies, the percentage of immune cells showed no significant association with genetic risk group (*P* = 0.27) or tumor size (*P* = 0.59) (Figs. 3A, 3B). Overall, there were no differences between risk groups regarding percentages of lymphocytes (*P* = 0.63) (Fig. 3C). In addition, enucleated tumors also did not show a significant difference between risk groups regarding lymphocyte density (*P* = 0.36) (Supplementary Fig. S5). On the other hand, within the lymphocyte population, the distribution of subsets varied significantly between risk groups, with low-risk tumors having a higher NK cell abundance (mean ± SD percentage of all lymphocytes: 62.1% ± 20.3% in low-risk tumors vs. 31.5% ± 24.5% in high-risk tumors; *P* = 0.0049), whereas high-risk tumors had more CD8 T cells (mean ± SD percentage of all lymphocytes: 39.5% ± 20.9% in high-risk tumors vs. 13.1% ± 17.1% in low-risk tumors; *P* = 0.021) (Fig. 3C, Supplementary Fig. S5).

### Infiltrating T Cells Are CD8 Dominant and Express Activation Markers

**Figure 4.**
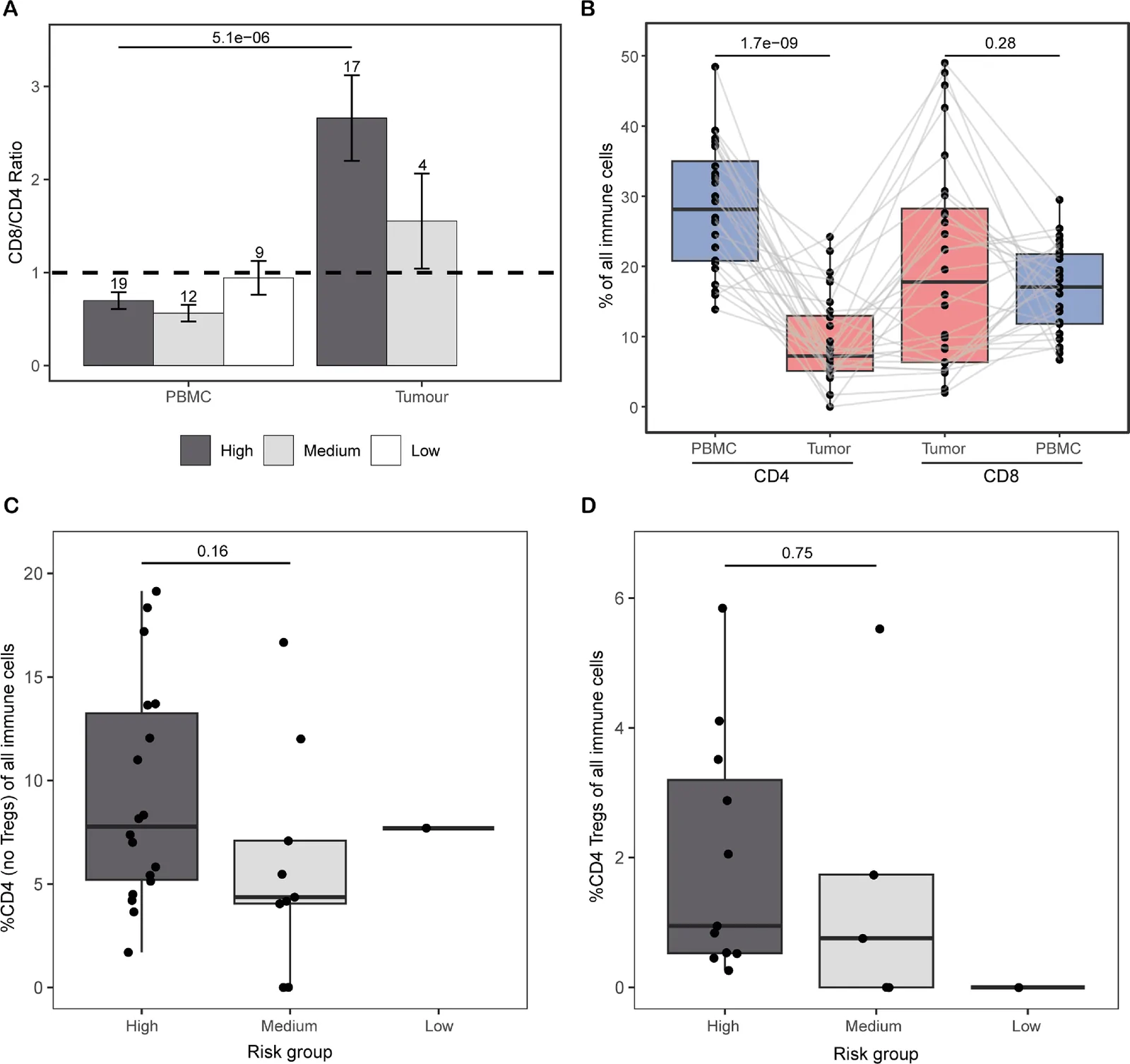
T-cell phenotype in tumor biopsies. (**A**) Bar plots showing the mean ratio of CD8 versus CD4 T cells in tumor biopsies and blood (PBMCs). Risk groups are indicated by shades of *gray*. *Error bars* show the standard error of the mean, and the number above each *error bar* indicates the number of samples that belong to that group. (**B**) Boxplot indicating patient-matched percentages of CD4 (left) and CD8 (right) T cells in blood (blue) and tumor (red) biopsies. Lines indicate observations from the same patient. (**C**, **D**) Boxplots showing the percentage of CD4 T cells (excluding regulatory T cells) (**C**) and regulatory T cells (**D**) of all immune cells in tumor biopsies.

Further characterization of the T-cell compartment was carried out to understand their presence and function in high-risk tumors. In the high-risk PUM tumors, a high intratumoral CD8/CD4 ratio was seen (mean 2.66 in tumor vs. 0.70 in blood; *P* = 5.1 × 10^−6^) (Fig. 4A). Medium- and low-risk tumors more frequently did not have sufficient amounts of T cells for the analysis of T-cell subsets than high-risk tumors (*P* < 0.01), again indicating that high-risk tumors were more T-cell infiltrated.

Further analysis of CD8 and CD4 T cells revealed that the high CD8/CD4 ratio in high-risk tumors was mostly caused by a lack of CD4 T-cell infiltration (*P* = 1.7 × 10^−9^), although some tumors also showed a higher level of CD8 T cells as compared to blood (Fig. 4B). CD4 T-cell abundance was not significantly different between risk groups for both non-regulatory CD4 T cells (*P* = 0.16) and CD127^–^CD25^+^ regulatory T cells (*P* = 0.75) (Figs. 4C, 4D).

**Figure 5.**
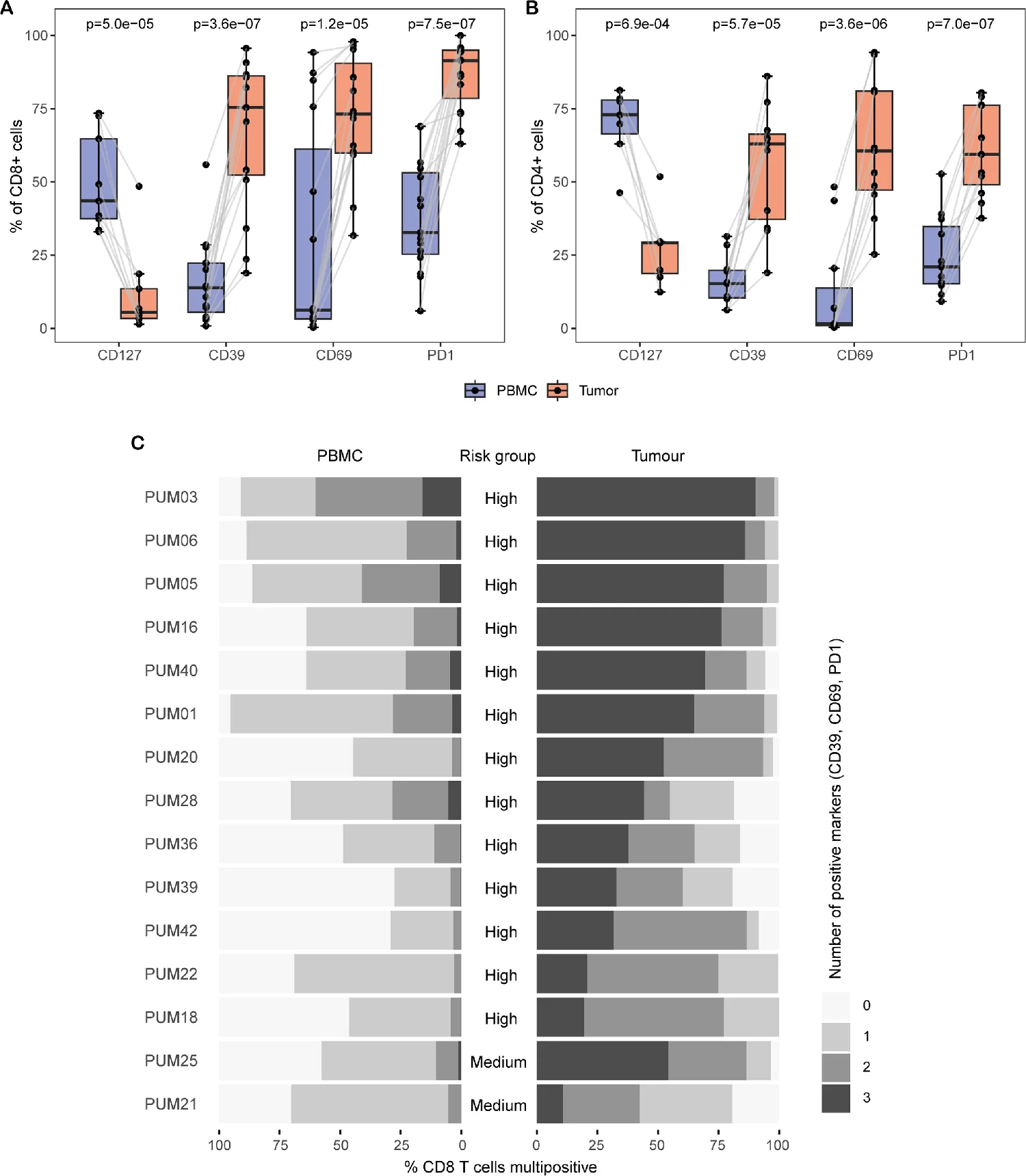
Marker expression on tumor-infiltrating T cells. (**A**, **B**) Boxplots showing the percentages of CD8 (**A**) and CD4 (**B**) T cells positive for the markers CD127, CD39, CD69, and PD-1. The data show each patient’s tumor biopsy and blood, indicated by the *gray* line. (**C**) Bars showing how many of CD8 T cells in patients were positive for none, one, two, or three of the markers CD39, CD69, and PD-1 in blood (*left*) and tumor (*right*). Text in the middle indicates the tumor risk group of the corresponding patient.

In an unbiased manner, we identified functional markers that show distinctly different expression in the tumor compared to circulating T cells (Supplementary Fig. S6A). In the tumor, expression of CD127, which is a marker for naïve and effector memory T cells, was strongly reduced. In contrast, activation markers CD39, CD69, and PD-1 were strongly upregulated on both CD8 and CD4 T cells in the tumor compared to the blood for all patients (Figs. 5A, 5B). As expected, both CD8 and CD4 T cells often expressed multiples of these three activation markers at the same time in the tumor (Fig. 5C, Supplementary Fig. S6B). Together, this showed that the T-cell phenotype in high-risk tumors was mainly activated CD8 T cells.

### Medium- and Low-Risk Tumors Are Characterized by NK Infiltration and Low HLA Class I Expression

**Figure 6.**
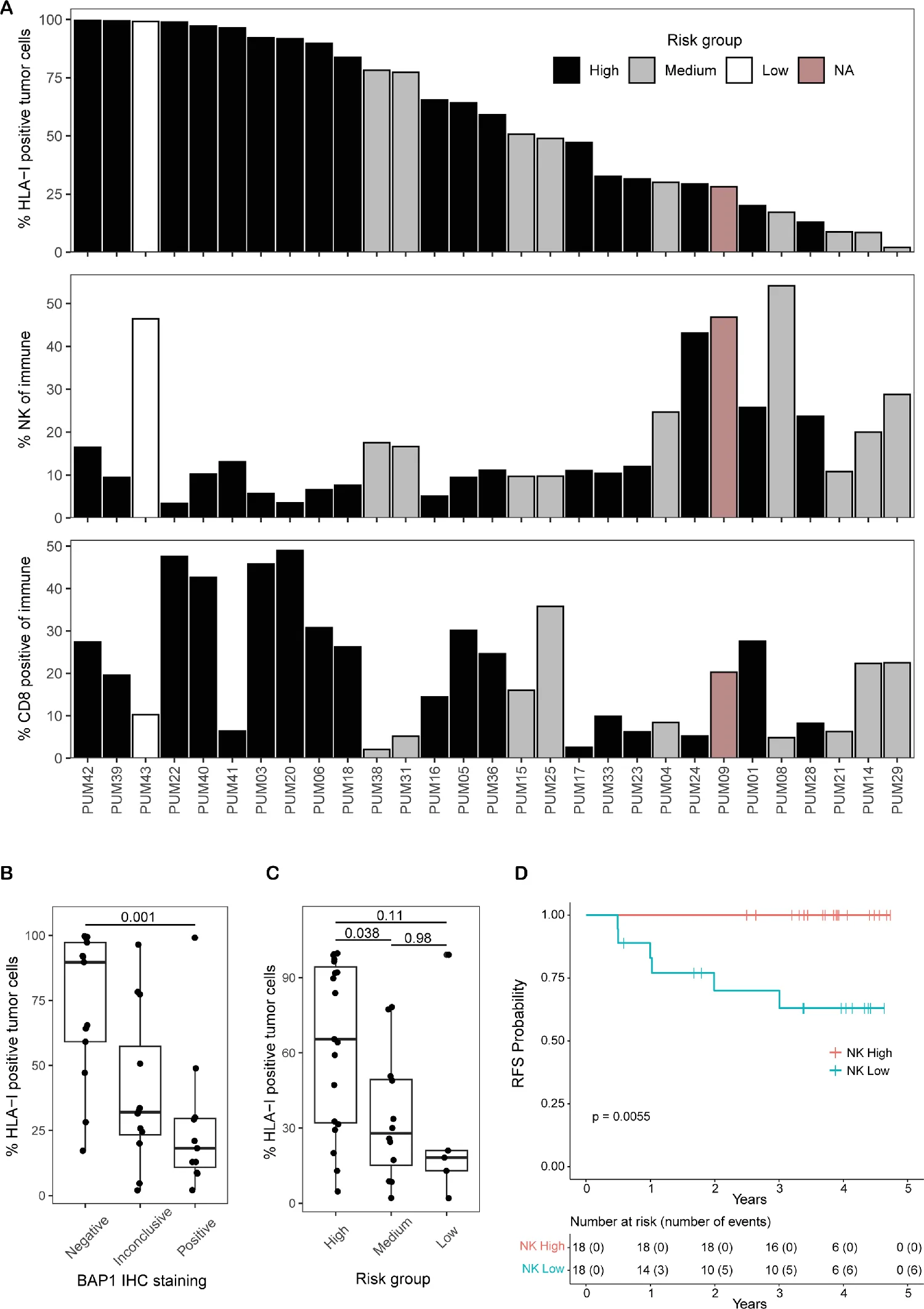
HLA class I expression inversely associates with intratumoral NK cell percentages. (**A**) Bar plots showing the percentages of HLA class I–positive tumor cells (*top*) and the NK cells (*middle*) and CD8 T cells (*bottom*) of all immune cells per patient. (**B**) Box plot showing the percentage of HLA class I–positive tumor cells versus the BAP1 staining that was performed on tissue. (**C**) Box plot showing the percentage of HLA class I–positive tumor cells versus the risk group. (**D**) Kaplan–Meier plot showing the RFS of two groups of patients, separated by the median percentage of NK cells into NK^high^ and NK^low^ groups.

Whereas T-cell infiltration was predominant in high-risk tumors, NK cells were the major lymphocyte subset in medium- and low-risk tumors (Fig. 3C). NK cells are known to have the capability to recognize a lack of HLA class I expression on cells.^38^ Indeed, tumors in which HLA class I expression was limited showed a higher relative presence of NK cells (Pearson's *r* = −0.42; 95% confidence interval [CI], −0.67 to −0.10) (Fig. 6A). Despite a higher presence of NK cells, their activation in the form of CCR7 or CD69 expression did not seem greatly enhanced (Supplementary Fig. S7A). NK cells also showed a negative correlation with CD8 T cells (Pearson's *r* = −0.43; 95% CI, −0.68 to −0.08) (Fig. 6A). CD8 T cells are known to bind to HLA class I. Correspondingly, CD8 T cells were more prevalent in tumors with high HLA class I expression (Fig. 6A). HLA class I has recently been found to be positively regulated by BAP1 in CM cell lines.^39^ BAP1 is located on chromosome 3 and therefore often lost in PUM. Strikingly, in our material, a strong negative correlation between BAP1 expression and HLA class I^+^ tumor cells was present (Fig. 6B). Similarly, HLA class I positivity was higher in higher risk groups (Fig. 6C). Interestingly, HLA class I expression above or below median did not hold any predictive value for tumor progression, although numbers of cases were limited (Supplementary Fig. S7B). In contrast, none of the patients with high NK cell abundance developed metastatic disease compared to six of the 18 patients with low abundance of NK cells in the tumor. (Fig. 6D). This is in agreement with the finding that NK cells were also mostly present in low- and medium-risk tumors (Supplementary Fig. S7C).

## Discussion

Thus far, the immune system in PUM has only been studied in enucleated eyes, thereby providing information only on a subset of all tumors in the current treatment era. Here, we explored the feasibility of TVRC biopsies from PUM to study tumor-infiltrating immune cells. This would allow the investigation of the immune system not only in the large PUMs that are enucleated, but also in small PUMs that are often treated with eye-conserving treatments.

Importantly, we were able to show that immune cells measured in tumor biopsies correlated with those found in enucleations, showing the reliability of this method. Additionally, in TVRC biopsies we validated several lymphocyte features that previously have been described only in enucleated eyes. We confirmed that high-risk tumors have more T-cell infiltration, dominated by CD8 T cells, as compared to low-risk tumors.^27^^,^^31^^,^^40^

Deeper characterization of membrane proteins on T cells showed certain markers to be specifically altered in the tumor as compared to blood. Fewer TILs expressed CD127, which is a marker for naïve and effector memory T cells and thus indicates differentiation of T cells in the tumor.^41^ Furthermore, the T-cell activation proteins CD39, CD69, and PD-1 showed higher expression in the tumor compared to the blood. Similarly, a recent study by Lucibello et al.^42^ found more CD39^+^PD-1^+^CD8 T cells inside the tumor compared to juxtatumoral tissue, indicative of intratumoral T-cell activation. However, they found minimal CD69 expression on CD8 T cells, which contrasts with the high intratumoral CD69 expression on CD8 T cells in our data. Yet, for CM, it has been reported that complete responders of adoptive cellular therapy had more stem-like CD39^–^CD69^–^CD8 T cells than non-responders.^43^ Hence, an adequate T-cell response requires a balance between activation and stemness status. CD8 T cells are often shown to correlate with poor genetic factors in PUM, but their prognostic value within certain risk groups is still unknown. TILs in PUM are activated but may be too terminally differentiated for a proper anti-tumor response. This would be in line with studies identifying high expression of lymphocyte activation gene 3 protein (LAG-3), an immune checkpoint associated with exhaustion, on TILs in PUM.^44^^,^^45^ Additionally, PD-1 and cytotoxic T-lymphocyte–associated protein 4 (CTLA-4) are well known immune checkpoint molecules, indicative of T-cell exhaustion. Although we showed intratumoral upregulation of PD-1 on TILs, reports on PD-1 and CTLA-4 are quite variable in PUM, stating both limited and high expression on transcriptional and protein levels.^31^^,^^40^^,^^45^^,^^46^

In addition to terminal differentiation or exhaustion, another explanation for ineffective tumor control by CD8 T cells could be the lack of antigens present in PUM. However, clonally expanded, antigen-specific T cells have been reported in subsets of patients.^45^^,^^47^ Antigens are presented on the cell membrane of tumor cells through HLA class I. In CM cell lines, HLA class I was reported to be positively regulated by BAP1 through histone modifications.^39^ However, in PUM, loss of BAP1 has shown to be associated with increased RNA expression of HLA class I.^48^ In addition, monosomy of chromosome 3, where BAP1 is located, has been shown to increase HLA class I levels.^29^ We also confirmed on a protein level that loss of BAP1 enhances HLA class I expression. This finding highlights the stark differences that are present between CM and PUM. In accordance with previous studies on PUM, we observed more HLA class I–expressing tumor cells in tumors dominated by CD8 T-cell infiltration.^27^ High HLA class I expression has also been associated with worse prognosis,^49^^,^^50^ which was not clear with regard to relapse-free survival (RFS) in our patient cohort, although the median RFS was not yet reached.

In tumors with limited HLA class I expression, we saw a higher relative abundance of NK cells, which is a relationship that has not been reported before in PUM patients. Similarly to our results, a high abundance of NK cells (defined by CD16^+^) has been found in TIL cultures of a single PUM tumor, although its risk group is unknown.^47^ NK cells have the ability to recognize a lack of HLA class I expression on tumor cells and kill them. Indeed, it has been shown that NK cells could efficiently lyse PUM cell lines in vitro*.*^38^^,^^47^ Strikingly, Neo et al.^51^ recently reported, based on pan-cancer The Cancer Genome Atlas (TCGA) analyses, that increased NK gene signatures correspond to higher risk of metastasis in uveal melanoma. This contrasts with our finding that patients with a higher percentage of intratumoral NK cells have improved relapse-free survival. It should be noted that NK gene signatures share great similarity with CD8 genes, such as *GZMA*, *GZMB*, and *TBX21*. Because our data showed a strict inverse balance between NK and CD8 T cells, this could influence TCGA analysis heavily. Another explanation could be that we looked at the relative abundance of NK cells and thus did not determine the absolute infiltration of NK cells. Early studies investigating TILs in PUM have shown limited absolute infiltration of NK cells in enucleations, which could diminish the importance of NK cells.^52^^,^^53^ However, one of those studies determined HLA expression and found HLA class I to be expressed in over 70% of all cells in their entire cohort.^52^ Those results are therefore not representative for low HLA class I–expressing PUMs, for which we found the NK cell abundance to be relatively high. It has been hypothesized that NK cell–mediated lysis of PUM cells may be quite effective in low HLA class I expressing PUMs, especially due to the low expression of HLA-G in PUM.^54^ Nevertheless, in vivo animal models have also shown that NK cells induce a pro–metastatic tumor phenotype.^51^ Altogether, this highlights that the role of NK cells in PUM is interesting but still poorly understood, making it an interesting population to further investigate.

There are also some limitations to the use of TVRC biopsies to study the tumor immune microenvironment. In contrast with high-risk tumors, many low-risk tumors had low numbers of TILs, meaning these could be characterized to only a limited extent. This problem may be addressed by optimizing the tissue dissociation method to improve the yield of immune cells or by taking larger biopsies. However, as low-risk tumors are often small, taking larger biopsies might not always be clinically feasible. TVRC biopsies are relatively unique for the Rigshospitalet in Copenhagen but are currently being adopted in more centers. This should improve the general knowledge on how to best use them for additional purposes, such as research.

Overall, we showed that TVRC tumor biopsies are an excellent source of PUM tissue, a finding that should enable research on potential tumor-related immune biomarkers in more PUM tumors, in particular thinner, low-risk tumors. We found an HLA class I–associated difference in the abundance of NK cells and T cells, as well as a correlation between NK cell infiltration and low disease recurrence. Further investigations into the tumor immune microenvironment of PUM are warranted to aid the development of successful treatments.

## Supplementary Material

Supplement 1
