# Supplementary material for: Transvitreal Retinochoroidal Biopsies of Primary Uveal Melanoma Reveal an Association of Low HLA Class I and High NK Cell Abundance in Low-Risk Disease
Source: Invest Ophthalmol Vis Sci. 2025 Feb 7;66(2):24. doi: 10.1167/iovs.66.2.24 (PMC11809448; doi:10.1167/iovs.66.2.24)
Supplement: Supplement 1 [file iovs-66-2-24_s001.pdf]

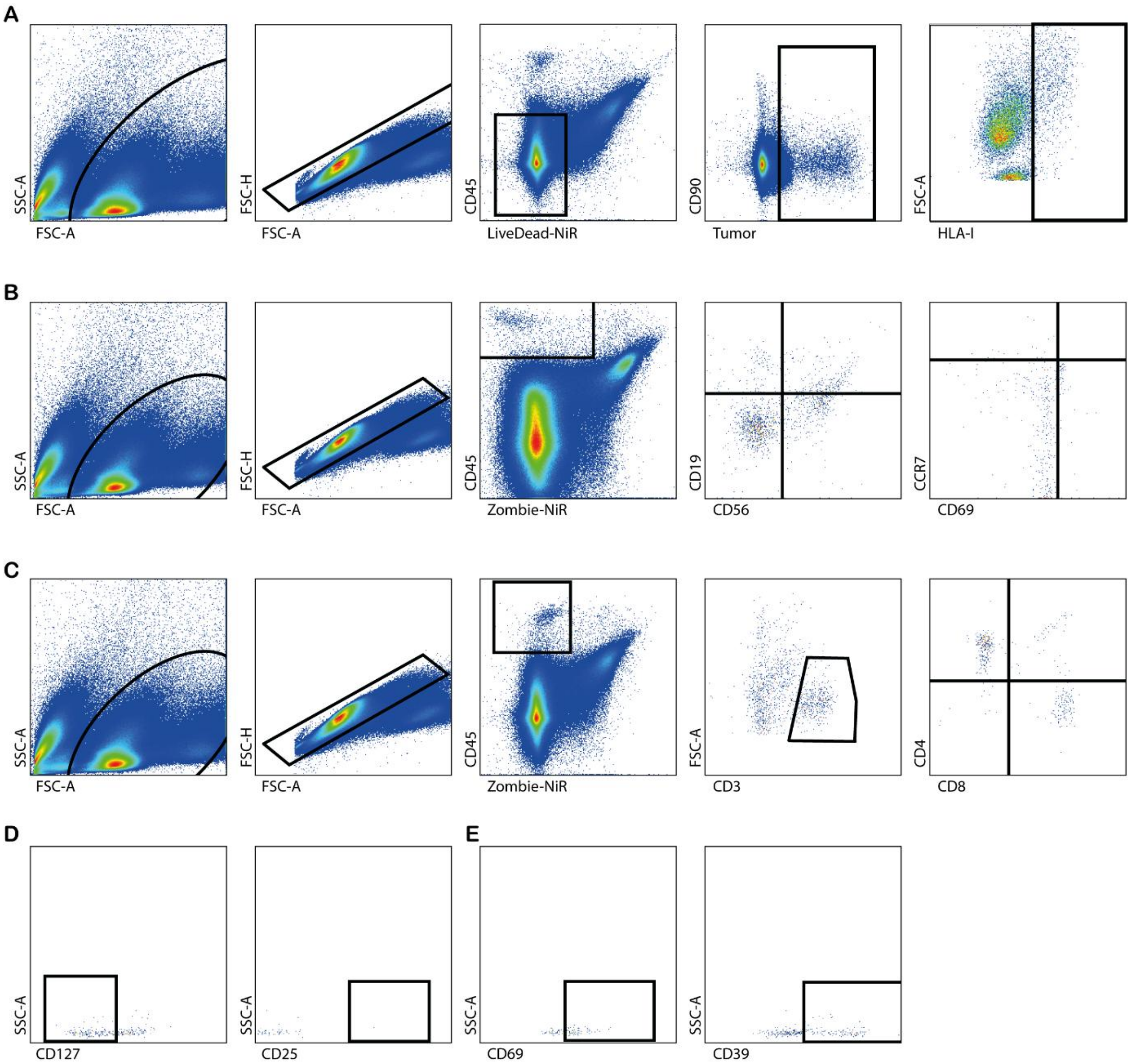

Supplementary Figure 1 Gating strategy of flow cytometry panels. **A-C** Basic gating involved cells based on FSC-A and SSC-A, followed by exclusion of doublets (FSC-A/FSC-H), and selection of live (LiveDead-NiR$^-$) immune cells (CD45$^+$) or live non-immune cells (CD45$^-$). **A** Tumor cells are gated using the tumor marker melanoma-associated chondroitin sulfate proteoglycan (MCSP), after which HLA class I expression was assessed. **B** B and NK lymphocytes were recognized based on single positivity for CD19 and CD56, respectively. CD56$^+$CD19$^-$ cells were then assessed for CD69 and CCR7 positivity. **C** T cells were gated on CD3, after which CD4 and CD8 single positive T cells were determined. **D** Within the CD4 population, regulatory T cells were determined based on negativity for CD127 and positivity for CD25. **E** Examples of gating of activation markers within CD4 and CD8 T cell populations, in this case for CD69 and CD39.

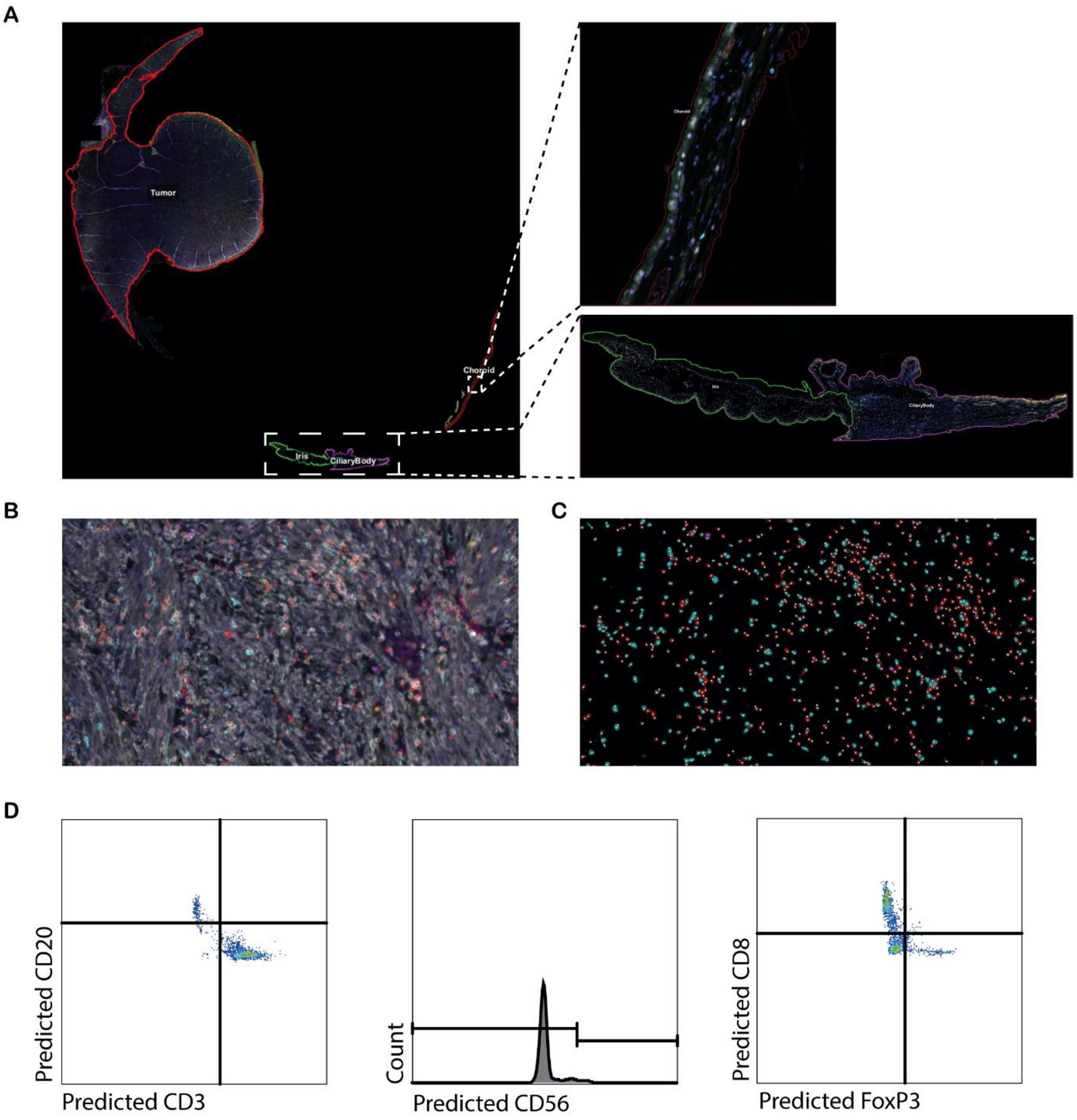

Supplementary Figure 2 Overview of mIHC analysis workflow **A** Overview of a staining of enucleated tissue, in which regions of interest (ROIs) can be drawn for tumor (red), choroid (dark red), iris (green), and the ciliary body (magenta). **B**,**C** (B) Zoomed in picture from panel A with (C) corresponding ImmuNet cell predictions. **D** ImmuNet predictions are saved as .fcs file per ROI and can then be gated in a similar way to flow cytometry experiments. Gating strategy is shown here, where cells are first divided in T cells ($CD3^+$) and B cells ($CD20^+$), after which NK cells ($CD56^+$) are gated from the double negative ($CD3^-CD20^-$) fraction. T cells are further subdivided in CD8 T cells ($CD8^+FoxP3^-$), CD4 (non-regulatory) T cells ($CD8^-FoxP3^-$), and regulatory T cells ($CD8^-FoxP3^+$).

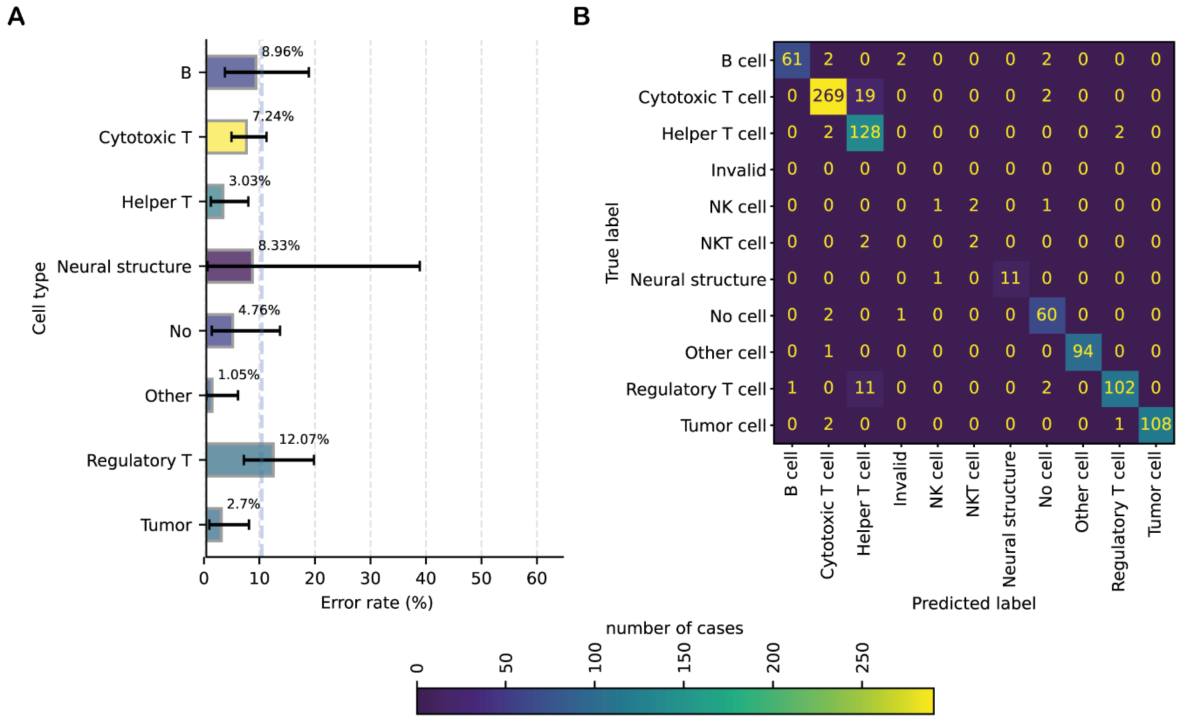

Supplementary Figure 3 Performance of ImmuNet for the mIHC dataset. **A** Error rate of the cell types that were detected by ImmuNet, based on manual annotations on the dataset. 95% Confidence intervals were computed using the Clopper-Pearson method. **B** Plot showing the interaction between manual cell type annotations (True label) against the ImmuNet predictions (Predicted label).

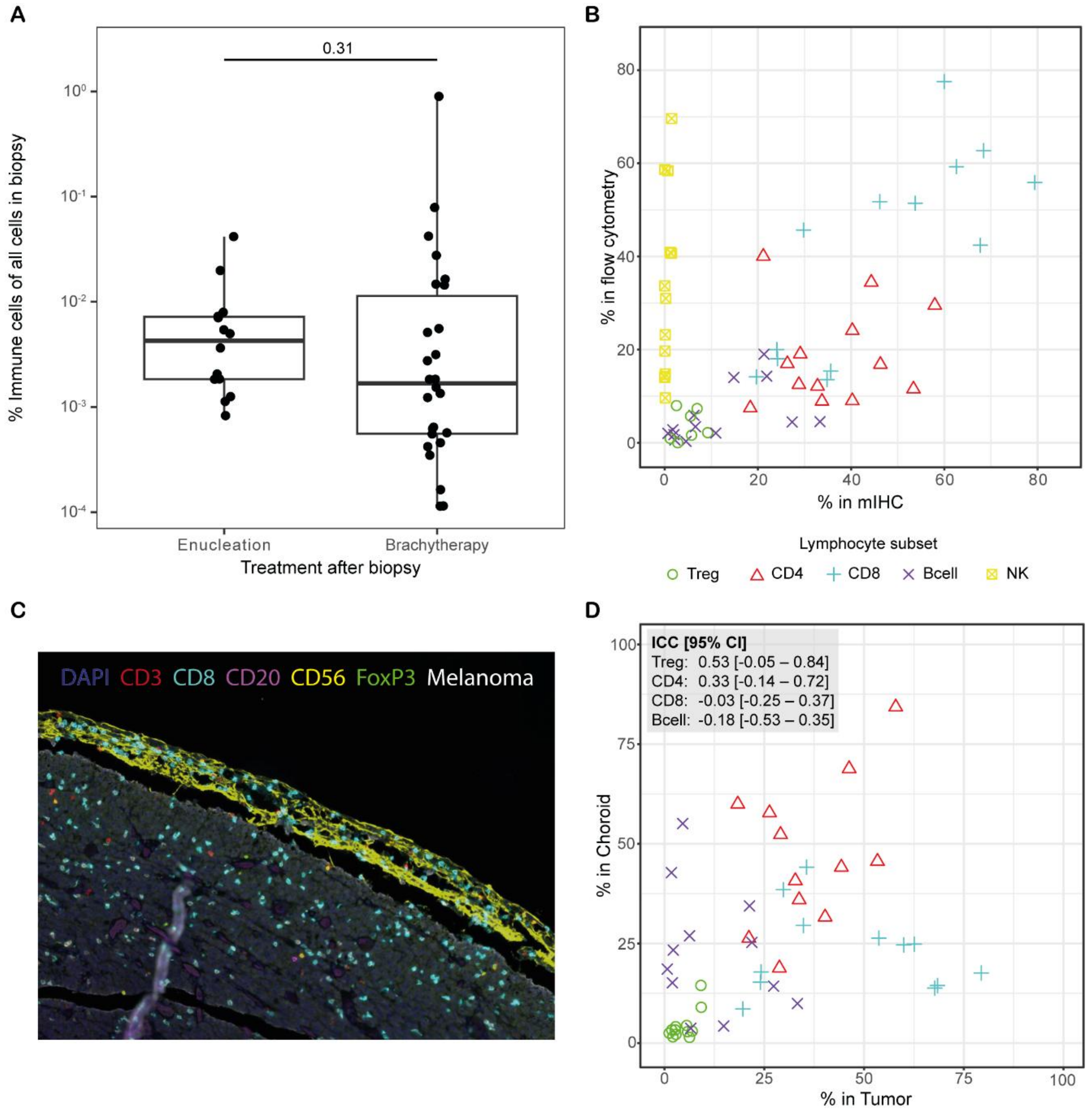

Supplementary Figure 4 Detection of immune cells in biopsies and enucleated samples **A** The percentage of immune cells in biopsies, measured with flow cytometry, dependent on local treatment. **B** Scatter plot showing the percentage of lymphocyte subsets in mIHC and flow cytometry data. Since NK cells could not be picked up sensitively enough by mIHC, NK cells were further left out from any analyses involving mIHC data. **C** mIHC image showing intense CD56 staining of the retina in yellow. **D** Scatter plot showing the percentage of lymphocyte subsets in the tumor and unaffected choroid within the same eye. Intra-class correlation coefficient (ICC) with 95% confidence interval (CI) was computed for each subset.

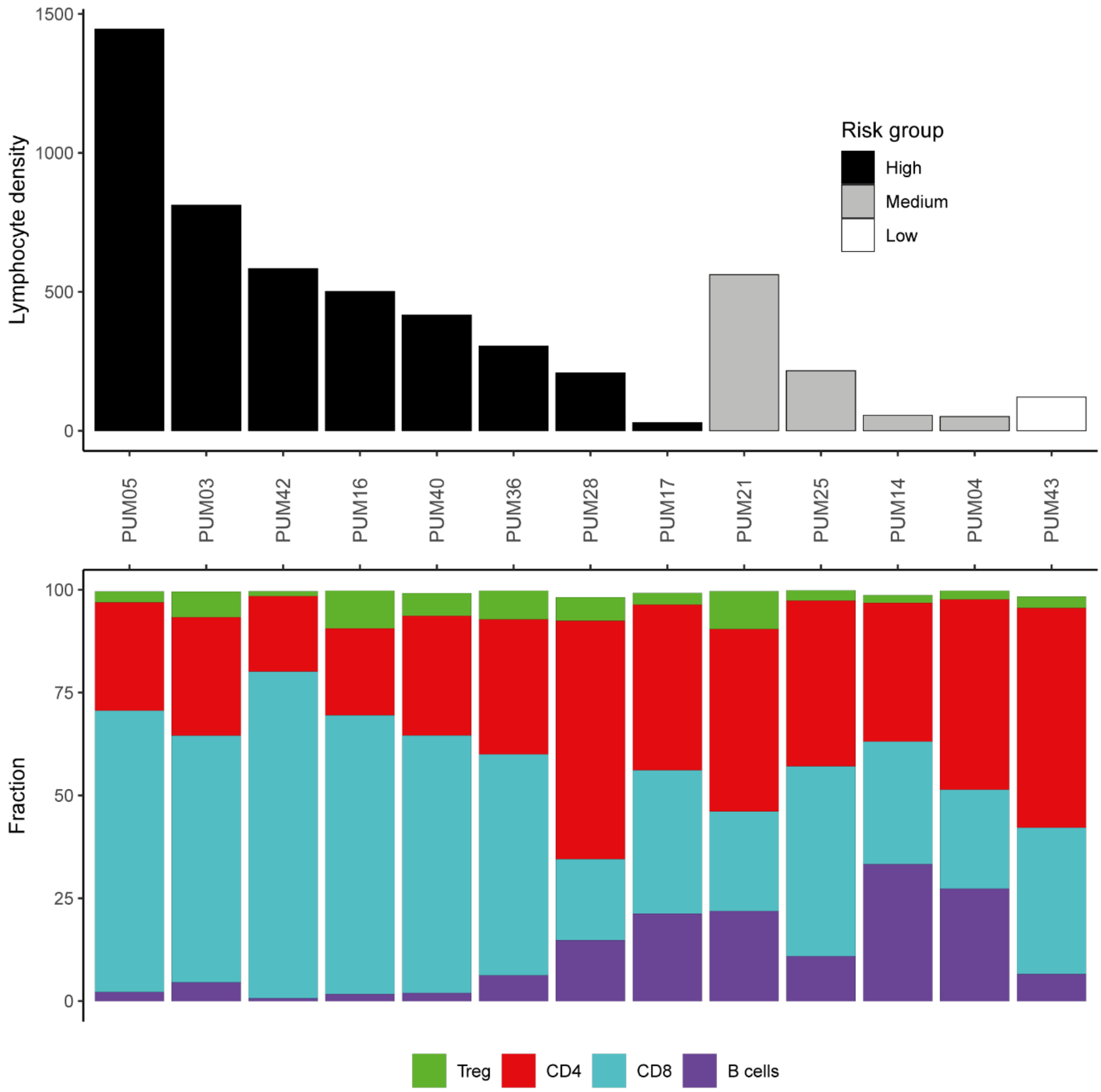

Supplementary Figure 5 Distribution of lymphocyte infiltration in mIHC staining of enucleated samples. The top plot shows a bar graph indicating the total density of tumor infiltrating lymphocytes per patient, sorted by risk group. The bottom plot shows the distribution of subsets of lymphocytes.

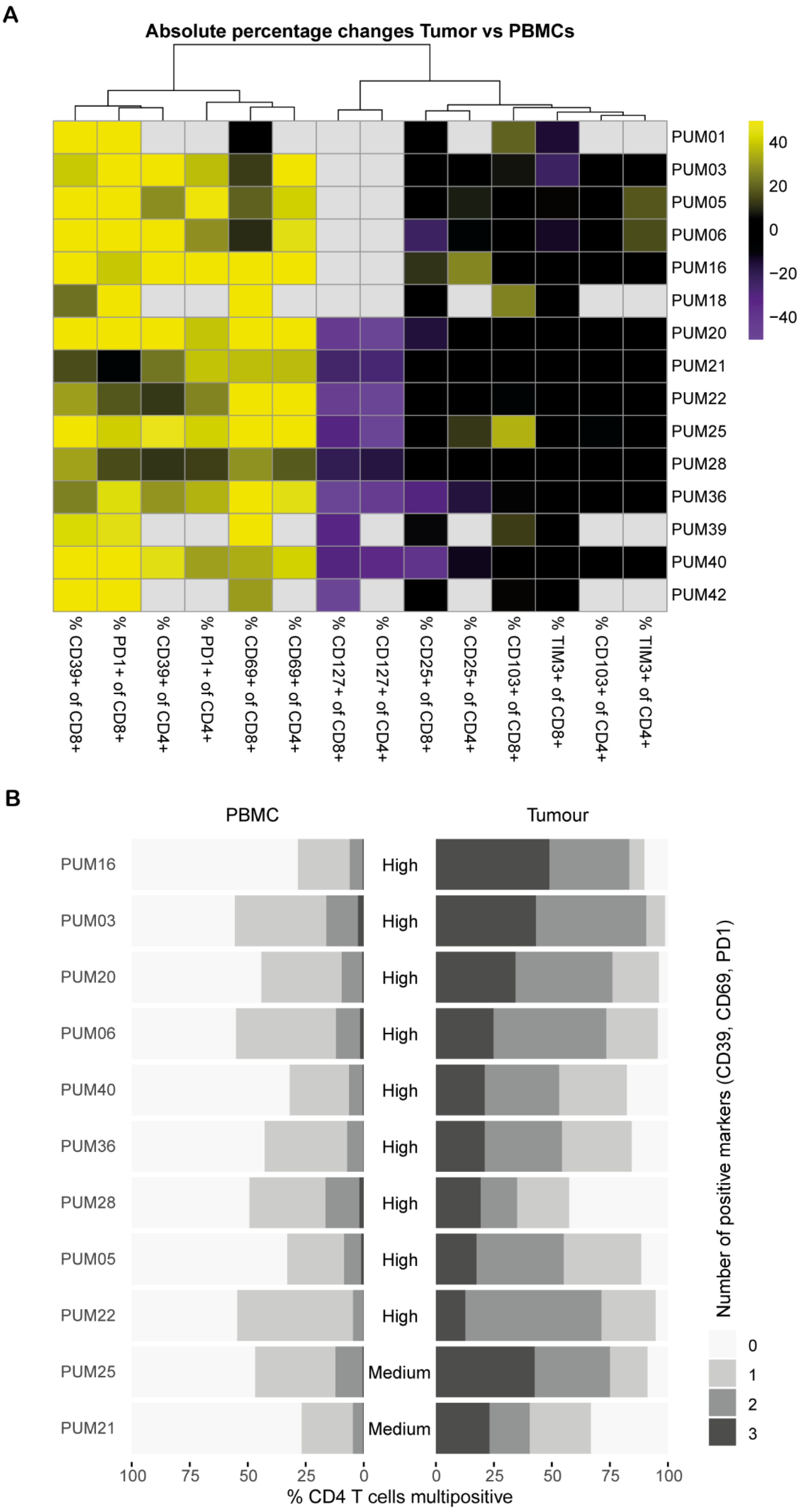
A
Absolute percentage changes Tumor vs PBMCs
PUM01
PUM03
PUM05
PUM06
PUM16
PUM18
PUM20
PUM21
PUM22
PUM25
PUM28
PUM36
PUM39
PUM40
PUM42
40
20
0
−20
−40
% CD39+ of CD8+
% PD1+ of CD8+
% CD39+ of CD4+
% PD1+ of CD4+
% CD69+ of CD8+
% CD69+ of CD4+
% CD127+ of CD8+
% CD127+ of CD4+
% CD25+ of CD8+
% CD25+ of CD4+
% CD103+ of CD8+
% TIM3+ of CD8+
% CD103+ of CD4+
% TIM3+ of CD4+
B
PBMC
Tumour
PUM16
PUM03
PUM20
PUM06
PUM40
PUM36
PUM28
PUM05
PUM22
PUM25
PUM21
High
High
High
High
High
High
High
High
High
Medium
Medium
100
75
50
25
0
0
25
50
75
100
% CD4 T cells multipositive
Number of positive markers (CD39, CD69, PD1)
0
1
2
3

Supplementary Figure 6 Marker expression on T cells measured by flow cytometry. **A** Heatmap showing patients in rows and percentages of markers on CD8 or CD4 T cells in columns. Colors indicate absolute differences in the percentage of positive cells for these markers in tumor tissue compared to healthy blood. Columns were clustered in an unsupervised manner according to similarity. Grey squares indicate missing or censored data due to not reaching cutoff values for the parent population. **B** Bars showing how many of CD4 T cells in patients are positive for 0, 1, 2, or 3 of the markers CD39, CD69, and PD-1 in blood (left) and tumor (right). Text in the middle indicates the tumor risk group of the corresponding patient.

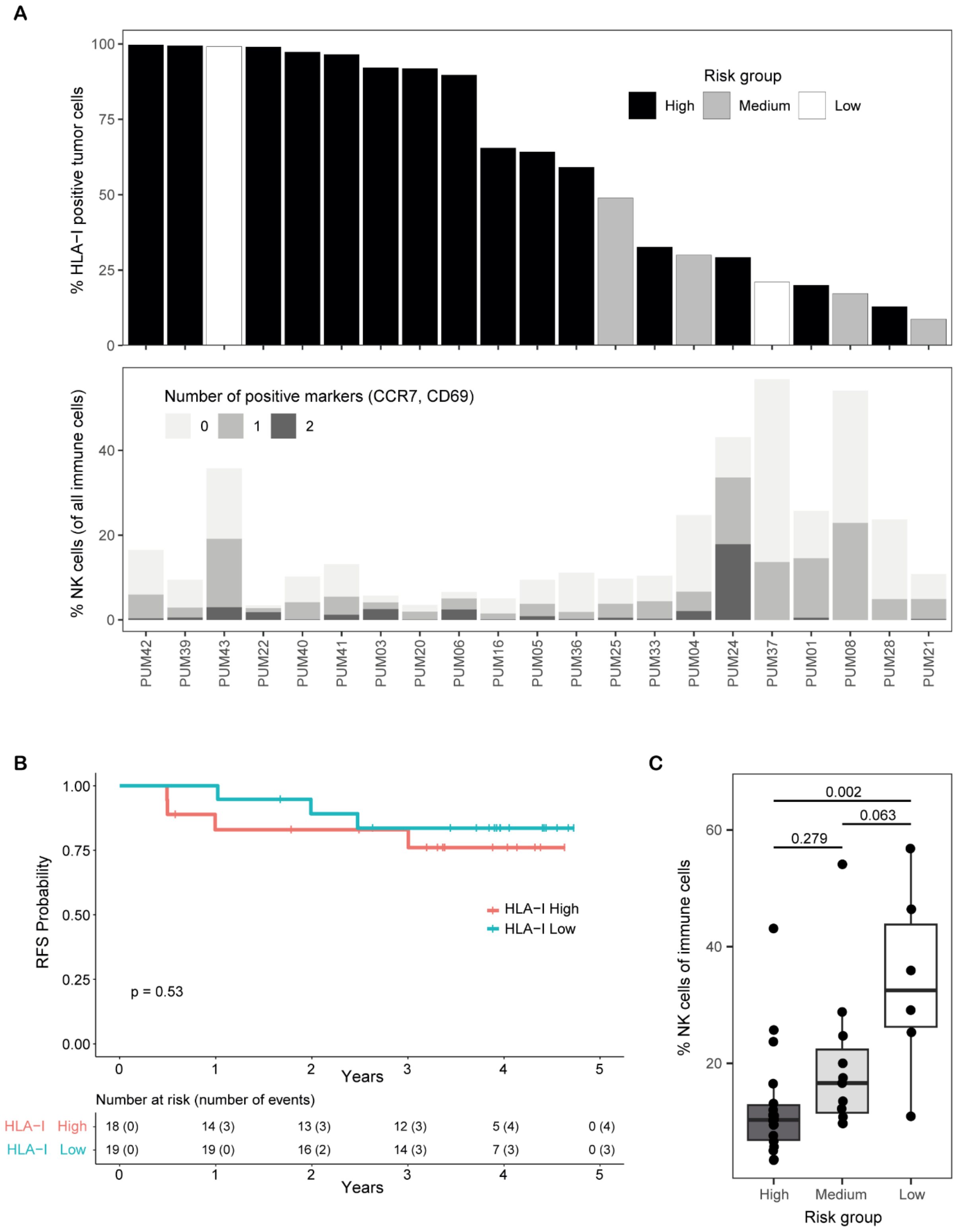

Supplementary Figure 7 NK cells and HLA class I expression in tumor biopsies. **A** Bar plot showing the percentage of tumor cells positive for HLA class I (top) and the distribution of markers CCR7 and CD69 on NK cells in the tumor (bottom) per patient. **B** Kaplan-Meier plot showing relapse-free survival (RFS) of two patient groups that have been separated into HLA-I^{high} and HLA-I^{low} based on the median percentage of HLA class I positive tumor cells. **C** Violin plot showing the percentage of NK cells of all immune cells in tumor biopsies for the different risk groups.

Supplementary Table 1. Flow cytometry antibodies

Target	Fluorochrome	Manufacturer	Clone	Catalog number	Panel		
CD3	BV786	BD Biosciences	SK7	563800	1		3
CD4	BV510	BD Biosciences	SK3	562970	1		3
CD8	BV605	BD Biosciences	SK1	564116	1		3
CD14[a]	SB702	Thermo Fisher Scientific	61D3	67-0149-41		2	
CD19	PE-Cy7	BD Biosciences	SJ25C1	557835		2	
CD25	APC-R700	BD Biosciences	2A3	565106			3
CD39	BV421	BD Biosciences	TU66	563679			3
CD45	BB700	BD Biosciences	HI30	746090	1	2	3
CD56	BV605	BD Biosciences	NCAM16.2	562780		2	
CD69	PE-Cy5.5	Thermo Fisher Scientific	CH/4	MHCD6918		2	3
CD90	AF700	Biolegend	5E10	328120	1		
CD103	PE	BD Biosciences	Ber-ACT8	550260			3
CD127[b]	BB515	BD Biosciences	HIL-7R-M21	564423			3
CD127[b]	VB FITC	Miltenyi Biotec	REA614	130-113-979			3
CD197 (CCR7)	BV421	Biolegend	G043H7	353208		2	
CD273 (PD-L2)	BV421	BD Biosciences	MIH18	563842	1		
CD274 (PD-L1)	PE-Cy7	BD Biosciences	MIH1	558017	1		
CD279 (PD-1)	PE-Cy7	BD Biosciences	EH12.1	561272			3
CD366 (TIM-3)	APC	Thermo Fisher Scientific	F38-2E2	17-3109-42			3
HLA-ABC	APC	BD Biosciences	G46-2.6	562006	1		
HLA-DR,DP,DQ	FITC	BD Biosciences	TU39	555558	1		
MCSP	PE	Miltenyi Biotec	REA1041	130-117-616	1		
LIVE/DEAD™ Fixable Near-IR Dead Cell Stain Kit	APC-Cy7	Thermo Fisher Scientific	-	L10119	1	2	3

[a]Due to retraction by the flow cytometer manufacturer of SB702 being a detectable fluorochrome this marker was excluded from analyses.

[b]Clone HIL-7R-M21 was used for PUM01 to PUM19, but did not end up working properly. Hence, analyses were only performed for clone REA614, which was used for PUM20 to PUM43.

Supplementary Table 2. Order and specifics of antibodies used for multiplex IHC staining

#	Target	Clone	Manufacturer	Catalog number	Dilution	Antigen retrieval	Opal color
1	CD56	MRQ-42	Cell Marque	156R-94	1/1500	EDTA	620
2	CD8	C8/144B	Dako	M7103	1/200	EDTA	690
3	CD20	L26	ThermoFisher	MS-340-S	1/300	EDTA	480
4	CD3	Sp7	ThermoFisher	RM-9107	1/200	EDTA	520
5	Foxp3	236A/E7	eBioscience Affymetrix	14-4777	1/100	EDTA	570
6	Melanoma mix<br>MART1<br>HMB45<br>Tyrosinase<br>SOX10	 A103<br>HMB45<br>T311<br>EP268	 Thermo Immunologic<br>Dako<br>Monosan<br>Cell Marque	 MS-799<br>M063401<br>MONX10591<br>383R	 1/300<br>1/600<br>1/200<br>1/500		780
